# Porous structure analysis of coconut shell–derived activated carbons prepared under different conditions

**DOI:** 10.1038/s41598-026-39432-4

**Published:** 2026-02-23

**Authors:** Mirosław Kwiatkowski, Xin Hu

**Affiliations:** 1 Department of Fuel Technology, Faculty of Energy and Fuels, AGH University of Krakow, al. Adama Mickiewicza 30, 30-059 Krakow, Poland; 2 Key Laboratory of the Ministry of Education for Advanced Catalysis Materials, Zhejiang Normal University, Jinhua, 321004 Zhejiang PR China

**Keywords:** Adsorption, Pores, Isotherms, Ammoxidation, Activation, Chemistry, Energy science and technology, Engineering, Materials science

## Abstract

This paper presents original results of the analysis of the influence of preparation conditions on the formation of the porous structure of activated carbons derived from coconut shells and doped with nitrogen by combining ammoxidation with potassium hydroxide chemical activation. The clustering-based adsorption analysis process, the quenched solid density functional theory, and the non-local density functional theory methods were used in the analyses. Based on the obtained results, a significant effect of both the activation temperature and the mass ratio of precursor to chemical activator on the formation of the porous structure of the prepared activated carbons was observed. The materials with the best adsorption properties were the activated carbons prepared at 700 °C with mass ratios of raw material to chemical activator of 3 and 4. These materials were characterised not only by the highest development of the microporous structure, as indicated by the *V*_hA_ values i.e.: 1.563 cm³/g and 1.542 cm³/g, respectively, but also by the lowest degree of surface heterogeneity, as suggested by the surface heterogeneity parameter *h* = 1.

## Introduction

One of the main problems facing the world today is the excessive emission of carbon dioxide (CO₂) into the atmosphere resulting from human activities^1^. Therefore, initiatives have been undertaken in highly industrialised countries to reduce CO₂ emissions^2^. Among others, technologies for CO₂ capture are being developed, with adsorption technologies considered the most promising^3–5^. The most effective adsorbents are activated carbons due to their significantly developed porous structure especially in the range of the smallest micropores, their very large specific surface areas, the high efficiency of the adsorption and the great possibilities for modifying their physicochemical properties^6^.

The increasing demands placed on activated carbons particularly for CO_2_ capture imply research into improving the adsorption properties of these materials to increase their ability to adsorb the aforementioned gas^7–9^. The CO₂ adsorption capacity of activated carbons can be effectively enhanced by doping with heteroatoms such as nitrogen^10,11^, sulphur^12–15^, phosphorus^16,17^, and boron^18,19^.

One of the most effective methods for this is ammoxidation, which involves the simultaneous oxidation and nitration of the precursor material^20^. This process aims to modify the surface of activated carbon by introducing nitrogen-containing functional groups that have an increased affinity for CO₂. This is due to the fact that carbon dioxide is an acidic gas and the nitrogen groups introduced on the surface of the activated carbon during ammoxidation are basic in nature^21^. This results in increased interaction between CO₂ and the carbon surface by: reacting nitrogen groups with CO₂, forming hydrogen bonds by CO_2_ with nitrogen-containing functional groups, and increasing the polarity of the activated carbon surface, which improves conditions favourable for the adsorption of polar molecules such as CO₂. The introduction of amino groups furthermore increases selectivity towards CO₂ even in the presence of other gases such as nitrogen (N₂), methane (CH₄), or oxygen (O_2_)^20,21^.

The efficiency of the ammoxidation process depends on its temperature, which is usually in the range of 300–500 °C. It should be noted that too low a temperature may not be sufficient to introduce the nitrogen groups and, in turn, too high a temperature may lead to degradation of the material structure. Furthermore, the efficiency of the ammoxidation process depends on the ammonia/oxygen ratio, with higher ammonia concentrations favouring the introduction of more nitrogen groups^20,21^. The ammoxidation process is also influenced by the duration of the process, i.e. a longer ammoxidation time may increase the number of functional groups, but may also lead to undesirable uncontrolled degradation of the carbon structure. The porous structure of activated carbon also has an influence on the ammoxidation process, i.e. material with a large specific surface area and a significantly well-developed porous structure in terms of micropores is better suited for modification by this method^20,21^.

## Materials and methods

The paper^21^ presents original research results dedicated to the development of a new method for the synthesis of nitrogen-doped porous activated carbons using coconut shells as a precursor. As part of the aforementioned research, coconut shells were cleaned, crushed and sieved to a grain size of 105–150 μm. The raw material thus prepared was then subjected to a carbonisation process in a quartz horizontal tube furnace at a nitrogen flow rate of 200 cm^3^/min. Samples were heated from room temperature to 500 °C at a heating rate of 5 °C/min.

The samples were successively held at the final temperature for 2 h and then cooled under a nitrogen atmosphere. For the chars obtained as described above, ammoxidation was carried out by mixing ammonia and air in a ratio of 1:10 at 350 °C for 5 h. The heating rate was 5 °C/min and the flow rate of the ammonia-air mixture was 100 cm^3^/min. Nitrogen-doped activated carbons (NC) produced under different conditions from coconut shells were designated NC-X-Y, where X is the activation temperature and Y is the ratio of KOH to NC.

Nitrogen adsorption isotherms at 196 °C were determined for the resulting nitrogen-doped activated carbons. Prior to the determination of these adsorption isotherms, the nitrogen-doped activated carbon samples were degassed under vacuum at 200 °C for a minimum of 12 h. On the basis of the determined nitrogen adsorption isotherms, the specific surface area of *S*_BET_ was calculated using the Brunauer-Emmett-Teller (BET) method^22^, the total micropore volume *V*_0_ using the t-plot method^23^, the total pore volume *V*_t_, and the micropore size distribution was determined using the Horvath-Kawazoe (HK) method^24^.

The properties of the raw material, as well as the production process methods and conditions determine the specific surface area, pore structure, and pore size distribution of the activated carbons. In particular, the ammoxidation process requires sufficiently precise control of temperature, pressure and process duration to obtain activated carbons with the expected adsorption properties. Therefore, the parameters of the porous structure must be precisely calculated from the determined adsorption isotherms in order to select the optimal preparation method and conditions.

The BET, t-plot and HK methods used in the aforementioned study have been criticised, however, for oversimplifications of the adsorption process models and the model pore structure and other important simplifying assumptions on which they are based. In particular, the BET model assumes that all adsorption sites have the same energy, which is not true for heterogeneous surfaces. Furthermore, the model of the adsorption process on which the BET method is based does not take into account the interactions between the adsorbate molecules^25^.

The BET method allows correct analysis results to be obtained only in a relatively narrow range of relative pressures, i.e. from 0.05 to 0.3 *P*/*P*_0_. In addition, the method is not suitable for the analysis of materials with a large number of micropores and, in particular, such as activated carbons or zeolites^25^.

The t-plot method is a popular method mainly used to study adsorption properties and pore structure, including especially the determination of specific surface area, micro pore volume and identification of pore type in porous materials^26^. The t-plot method is based on comparing the adsorption isotherm of the material under investigation with a reference isotherm, the reference curve, which represents adsorption on a non-porous surface with similar properties, and *t* is the thickness of the adsorbate layer on the reference surface^26^.

The t-plot method assumes that adsorption on a non-porous surface proceeds in a similar way to adsorption on the surface under investigation, until the pores are filled. If, on the other hand, the t-plot is linear and passes through zero, this means that the material has no micropores and all adsorption occurs only on the outer surface^26^. Unfortunately, the t-plot method has many disadvantages due to the simplifying assumptions made. In particular, the t-plot method assumes that the surface of the material is energetically homogeneous, which, when analysing activated carbons whose surfaces are mostly heterogeneous, can lead to significant errors in the interpretation of the results^26^. Furthermore, the results of the t-plot method depend significantly on the choice of an appropriate reference curve, and an inappropriate choice of reference curve can lead to erroneous conclusions regarding pore structure^26^.

The Horvath-Kawazoe (HK) method used to determine pore size distributions assumes that micropores have the shape of parallel slits^27^. In reality, however, many porous materials have pores that are, for example, cylindrical or spherical in shape, with the consequence that adopting a pore model that deviates from reality can lead to erroneous results^28^. If cylindrical pores are present in the material, the HK method usually overestimates the actual pore width.

The HK method is based on the Lennard-Jones potential model, which does not take into account actual energy effects and thus does not always accurately describe adsorbate-adsorbent interactions, but in reality adsorption interactions are more complex^29^. This method assumes that the surface of the adsorbent is perfectly homogeneous and thus does not take into account the influence of chemical and structural heterogeneity of the surface, which is an oversimplification for most activated carbons^30^. Furthermore, the HK method does not take into account capillary condensation in micropores, making it unsuitable for mesopore analysis^31^. The results obtained with the HK method also vary depending on the adsorbate used and the temperature at which the adsorption isotherms are determined. It is further pointed out that, although the HK method is based on the potential of adsorbate-adsorbent interactions, it neglects entropic effects for small adsorbate molecules^32^. In addition, network effects are not taken into account if the adsorbate particles used in the analysis are smaller than the pore diameters, resulting in an overestimation of the calculated pore widths^32^.

In view of the above, the calculation results obtained using the aforementioned methods, and thus at the same time the information obtained on the analysed structure, are subject to significant error and a high degree of uncertainty, therefore, a new unique series of studies has been carried out on the analysis of the porous structure of activated carbons using the advanced method of the clustering based adsorption analysis process (LBET) method^33,34^, the non-local density functional theory (NLDFT) method^35^, and the quenched solid density functional theory (QSDFT) method^36^. Considerable attention was paid to the complementarity of the methods used as well as the reliability of the results obtained.

The LBET method, a detailed description of which was presented in earlier work^33,34^, is derived from the original universal theory of adsorption and the models derived from it for the adsorption process on the surfaces of homogeneous carbonaceous adsorbents, which are a significant development of the Langmuir (L)^37^ and Brunauer-Emmett-Teller (BET) adsorption models^22^. According to the adsorption model adopted in the LBET method, adsorbate molecules adsorb into pores to form clusters of adsorbate molecules, the size of which is limited by the size and shape of the pores or the competitive expansion of neighbouring clusters. Adsorbate molecules adsorbed as a result of interactions between the adsorbent and the adsorbent surface are treated as the first adsorption layer, and adsorbate molecules attaching to already adsorbed molecules are treated as the second and subsequent adsorption layers. In contrast to the BET theory, the interaction of second and subsequent layer molecules with the adsorbent surface is not excluded at the same time. The LBET method also takes into account the interactions between molecules adsorbed on different layers. In this method, a numerical procedure for the fast multivariate identification of adsorption systems based on vapour and gas adsorption isotherms is implemented, which is also used to determine the shape of the adsorption energy distribution on the first layer and to determine the value of the surface heterogeneity index *h*^33,34^. The reliability of the identification of adsorption systems by the LBET method is assessed based on the dispersion of the errors in fitting the theoretical adsorption isotherms to the experimental isotherms *σ*_*e*_ and the quality index of the identification of adsorption systems *w*_id_^33,34^.

One of the most important pieces of information in the characterisation of porous adsorbents is the pore size distribution, and a number of methods have been developed in the last few decades to determine these distributions from adsorption isotherms, i.e. Horvath-Kawazoe^27^ and a group of methods derived from the density functional theory (DFT), which are currently the most popular^35,36^. Research into the application of the aforementioned density functional theory to the analysis of adsorption processes was first carried out by Evans and Tarazona^38^, but it was not until Seaton et al.^39^ used the DFT method to calculate the pore size distribution. These authors assumed that the measured adsorption isotherm was the sum of the isotherms of the individual pores, however, this approach was inaccurate especially in the micropores range.

Significant improvements in accuracy have only been achieved using the non-localized density functional theory (NLDFT) method^40^, which has become very popular and has proven particularly successful in the description of ordered silicates and zeolites. In the case of activated carbons, however, the application of the NLDFT method is problematic due to the adoption of a model of independent fractured pores with ideal graphitic walls^41^. Furthermore, the NLDFT method assumes a chemically and geometrically ideal smooth surface, disregarding the chemical and geometric heterogeneities, as well as deviations from the ideal model pore structure, of real microporous materials^42^. However, most adsorbents have molecularly rough microporous surfaces, so the consequence of this discrepancy between the theoretical assumption of smooth and homogeneous surfaces and the heterogeneity of real adsorbents at the molecular scale is that the theoretical NLDFT adsorption isotherms show multiple steps. These stages are related to the layer transition occurring with the formation of a monolayer, a second adsorbed layer and subsequent layers.

The above-mentioned problem is particularly evident in the case of microporous carbonaceous materials with surfaces that are highly heterogeneous both chemically and geometrically, which furthermore exhibit wide pore size distributions, and it manifests itself in the occurrence of distortions in the pore size distributions in the form of artificial breaks in the calculated pore size distributions. In recent years, attempts have been made to move away from simplified models of the structure and surface of porous materials, as the assumed model pore shape has been shown to strongly influence the characterisation results of these materials^43^.

Among others, Ravikovitch and Neimark^44^ proposed a new model called QSDFT, taking into account surface heterogeneities in one dimension, which allowed a significant improvement in the agreement between experimental and theoretical adsorption isotherms, especially in the low pressure range. This significantly increased the reliability of the determined pore size distributions and eliminated the artificial gaps in pore size distributions present with the NLDFT method^44,45^. It should be mentioned that with the QSDFT method, in addition to determining the pore size distributions, it is also possible to determine the micropores specific surface area *S*_QSDFT_, and the volume of micropores *V*_QSDFT_.

## Discussion of the results

**Fig. 1:**
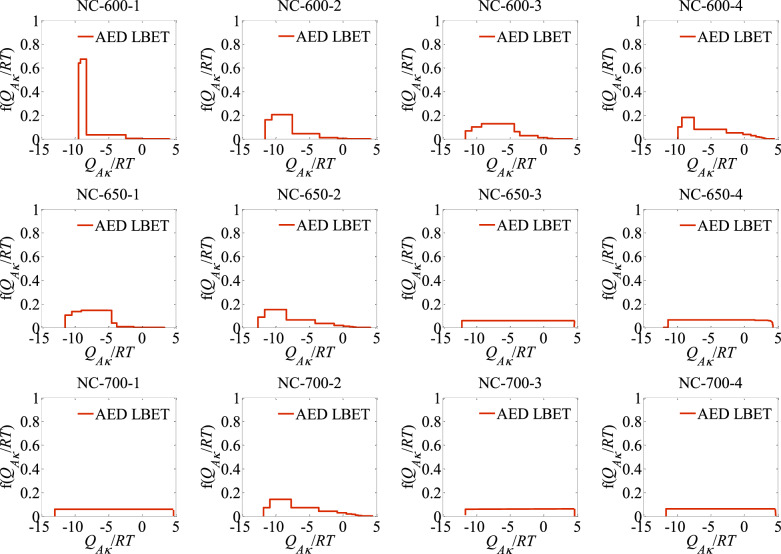
The adsorption energy distributions (AED LBET) obtained via LBET method for the all analysed activated carbons.

**Fig. 2:**
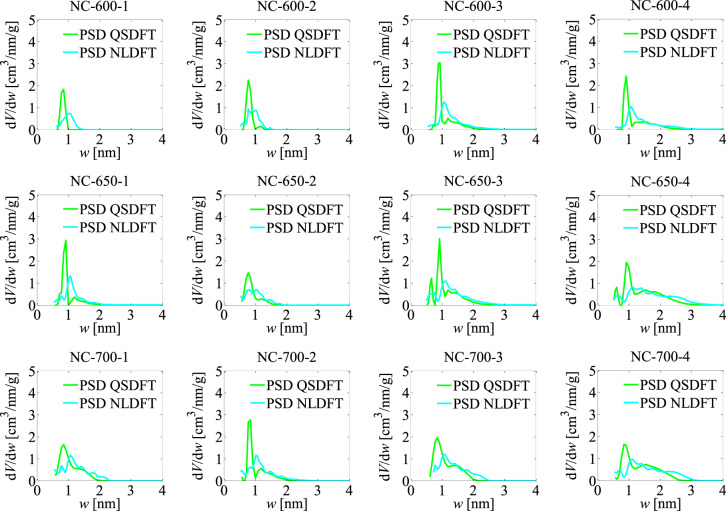
The pore size distribution plots for the all analysed active carbons obtained via the QSDFT and NLDFT methods.

The results of the analyses carried out using the LBET, and QSDFT as well as NLDFT methods are gathered in Table 1 and respectively in Table 2. In addition, Fig. 1 shows plots of the adsorption energy distribution determined by the LBET method, while Fig. 2 presents the pore size distributions determined using the QSDFT and NLDFT methods based on nitrogen adsorption isotherms.

**Table 1:** The results of the analysis of a porous structure of activated carbons prepared from coconut shells based on nitrogen adsorption isotherms, using the LBET method.

Sample	Model No.	*V*_hA_ [cm^3^/g]	*α*	*β*	*Q*_A_/*RT*	*B*_C_	*h*	*σ*_e_	*w*_id_
NC-600-1	7	0.322	0.25	1.00	− 9.43	7.71	5	0.054	0.67
NC-600-2	13	0.434	0.35	2.32	− 11.56	7.73	9	0.083	0.54
NC-600-3	15	0.743	0.51	1.00	− 11.63	7.62	9	0.089	0.66
NC-600-4	25	0.672	0.75	1.00	− 9.87	7.69	7	0.058	0.71
NC-650-1	15	0.556	0.28	1.00	− 11.43	3.65	9	0.089	0.73
NC-650-2	13	0.523	0.62	1.00	− 12.64	5.94	9	0.087	0.65
NC-650-3	2	1.328	0.00	1.00	− 12.18	7.78	1	0.021	0.79
NC-650-4	25	1.449	1.00	1.00	− 11.99	5.68	7	0.054	0.72
NC-700-1	2	1.320	0.00	1.04	− 12.96	7.93	1	0.023	0.81
NC-700-2	28	0.757	0.71	1.00	− 11.82	7.81	9	0.085	0.68
NC-700-3	2	1.563	0.00	1.00	− 11.95	7.79	1	0.018	0.82
NC-700-4	2	1.542	0.86	1.00	− 11.62	7.83	1	0.019	0.83

Where: Model No. is the number of the best fitted LBET class model, *V*_hA_ is the volume of the first adsorbed layer, *α* is the geometrical parameter of the porous structure determining the height of the adsorbate molecule clusters; *β* is the geometrical parameter of the porous structure determining the width of the adsorbate molecule clusters, *Q*_A_/*RT* is the dimensionless adsorption energy parameter for the first adsorbed layer; *B*_C_ is the adsorption energy parameter for the higher adsorbed layers; *h* is the surface heterogeneity parameter; *σ*_e_ is the dispersion value of the fit error, and *w*_id_ is the is the identifiability index.

**Table 2:** The results of the analysis of a porous structure of activated carbons prepared from coconut shells based on nitrogen adsorption isotherms, using the QSDFT and NLDFT methods.

Material	*S*_QSDFT_ [m^2^/g]	*V*_QSDFT_ [cm^3^/g]	*S*_NLDFT_ [m^2^/g]	*V*_NLDFT_ [cm^3^/g]
NC-600-1	790	0.326	690	0.317
NC-600-2	1155	0.530	1011	0.519
NC-600-3	1434	0.757	1221	0.734
NC-600-4	1201	0.682	1006	0.660
NC-650-1	1221	0.584	1040	0.565
NC-650-2	1374	0.629	1220	0.621
NC-650-3	1838	0.942	1579	0.914
NC-650-4	1992	1.239	1713	1.202
NC-700-1	2005	0.909	1763	0.885
NC-700-2	1687	0.850	1465	0.831
NC-700-3	2082	1.066	1815	1.032
NC-700-4	2108	1.197	1810	1.159

Where: *S*_QSDFT_ is the micropores specific surface area, and *V*_QSDFT_ is the volume of micropores obtained via QSDFT method; *S*_NLDFT_ is the micropores specific surface area, and *V*_NLDFT_ is the volume of micropores obtained via NLDFT method.

Based on the results obtained, it can be concluded that the NC-600-1 activated carbon is characterized by constraints on nitrogen cluster expansion, likely due to the influence of neighbouring clusters. This is supported by the best-fitted model number within the LBET class. The value of the parameter *V*_hA_ indicates a poor texture development (*V*_hA_ = 0.322 cm^3^/g), and the determined values of the parameter *α* and *β* indicate that low and non-branching clusters of nitrogen molecules are formed in the pores of the NC-600-1 sample (*α* = 0.25, and *β* = 1.00).

The values of the adsorption energy parameters *Q*_A_/*RT*, and *B*_*C*_ indicate favourable conditions for the multilayer adsorption process (*Q*_A_/*RT* = − 9.43, and *B*_C_ = 7.71), and the surface of this material is characterized by a high degree of heterogeneity (*h* = 5). On the other hand, the values of the structural parameters, i.e. the size of the specific surface area and the pore volume determined by the QSDFT and NLDFT methods (Table 2), confirm the results obtained with the LBET method, indicating a poor development of the porous structure (*S*_QSDFT_ = 790 m^2^/g, *V*_QSDFT_ = 0.326 cm^3^/g, *S*_NLDFT_ = 690 m^2^/g, *V*_NLDFT_ = 0.317 cm^3^/g).

The shape of the adsorption energy distribution obtained via the LBET method (AED LBET) indicates a dominant proportion of adsorption sites with a narrow range of adsorption energies and a smaller proportion of adsorption sites with a wide energy range (see Fig. 1).

In turn, the shape of the pore size distributions determined by the QSDFT method indicates a dominant share of pores with sizes below 1 nm, which is consistent with the results obtained using the LBET method. Note that the pore size distribution determined by the NLDFT method shows a peak shift towards larger micropores, which is due to the different pore model adopted in this method. Subsequently, the activated carbon NC-600-2, produced at a mass ratio of 2, was analyzed and is characterized by a larger volume of the first adsorbed layer (*V*_hA_ = 0.434 cm^3^/g) compared to sample NC-600-1. Low but significantly branching clusters of nitrogen molecules form in the pores of this material (*α* = 0.35, and *β* = 2.32), and its surface is highly heterogeneous (*h* = 9).

The values of specific surface area as well as pore volume determined by QSDFT and NLDFT methods indicate a greater development of the microporous structure of this sample compared to NC-600-1 (see Table 2). Analysis of the shape of the adsorption energy distribution for sample NC-600-2, showed a predominant proportion of sites with high adsorption energy, but less compared to the sample designated NC-600-1. In contrast, the pore size distribution indicate a wider range of pore sizes, which correlates with the results obtained using the LBET method.

The next activated carbon sample, NC-600-3, exhibits similar constraints in nitrogen molecule cluster expansion. The parameter *V*_hA_ for this sample is the highest among those obtained at an activation temperature of 600 °C (*V*_hA_ = 0.743 cm^3^/g). Medium-height clusters of adsorbate molecules form in the pores of this material. The values of the adsorption energy parameters and the surface heterogeneity parameter are virtually identical to those of sample NC-600-2.

For sample NC-600-3, the highest i.e. structure parameter values were determined among activated carbons obtained at 600 °C: the micropores specific surface area, and the volume of micropores obtained via QSDFT, and NLDFT methods (*S*_QSDFT_ = 1434 m^2^/g, *V*_QSDFT_ = 0.757 cm^3^/g, *S*_NLDFT_ = 1221 m^2^/g, *V*_NLDFT_ = 0.734 cm^3^/g). The shape of the adsorption energy distribution determined for this sample indicates a wide range of adsorption energies, and the shape of the pore size distribution determined by QSDFT and NLDFT methods indicates the dominant contribution of micropores to the total porosity of this sample.

When analysing the results obtained for the NC-600-4 activated carbon, a distinct mechanism for limiting the expansion of nitrogen molecule clusters becomes apparent, one that is related to the geometric constraints of the pores. The value of the calculated parameter *V*_hA_ for this sample is noticeably smaller (*V*_hA_ = 0.672 cm^3^/g) than the value of this parameter obtained for the NC-600-3 sample. Large clusters of nitrogen molecules form in the pores of the NC-600-4 material (*α* = 0.75, and *β* = 1.00), and its surface is characterised by significant heterogeneity (*h* = 7).

The values of the porous structure parameters determined for sample NC-600-4, indicate a lower development of microporosity compared to activated carbon NC-600-3 (*S*_QSDFT_ = 1201 m^2^/g, *V*_QSDFT_ = 0.682 cm^3^/g, *S*_NLDFT_ = 1006 m^2^/g, *V*_NLDFT_ = 0.660 cm^3^/g). The shape of the adsorption energy distribution (AED LBET) in turn indicates a narrower range of adsorption energies on the surface of this material, and the pore size distribution in turn indicates a greater proportion of small mesopores in the total pore structure.

The next activated carbons analysed were samples obtained at an activation process temperature of 650 °C. Sample NC-650-1 is characterised by limitations on the extension of nitrogen molecule clusters due to the competitive extension of neighbouring clusters and the average volume of the first adsorbed layer (*V*_hA_ = 0.556 cm^3^/g). In the pores of this material, low and non-branching clusters of adsorbate molecules are formed (*α* = 0.28, and *β* = 1.00), and the values of the adsorption energy parameters indicate conditions favouring multilayer adsorption (*Q*_A_/*RT* = −11.43, and *B*_C_ = 3.65). The magnitude of the specific surface area and pore volume determined by the NLDFT and QSDFT methods showed a significant development of microporosity in the sample analysed (see Table 2).

The NC-650-2 activated carbon sample obtained at a mass ratio precursor to KOH of 2 differs significantly in the greater development of the porous structure compared to the NC-650-1 sample, as indicated by the greater height of the clusters of nitrogen molecules forming in its pores and the size of the specific surface area and pore volume (see Table 1). The shape of the adsorption energy distribution determined for this sample is characterised by only a slightly higher proportion of high adsorption energy sites compared to sample NC-650-1.

The NC-650-3 sample, which was analysed next, is already characterised by a more developed pore structure, as indicated by the significantly higher value of the *V*_hA_ parameter (*V*_hA_ = 1.328 cm^3^/g), and the values of the geometrical parameters indicate the adsorption of single nitrogen molecules on the surface of this material (*α* = 0.00, and *β* = 1.00). The significant development of the porous structure of NC-650-3 activated carbon is also confirmed by the values of the determined structure parameters, i.e. *S*_QSDFT_, *V*_QSDFT_, *S*_NLDFT_, *V*_NLDFT_ (see Table 2). The shape of the adsorption energy distribution determined for this sample, in turn, indicates a uniform distribution of adsorption energy on its surface.

The NC-650-4 activated carbon analysed next, is also characterised by a significant *V*_hA_ parameter value (*V*_hA_ = 1.449 cm^3^/g), and very high and unbranched clusters of nitrogen molecules form in its pores (*α* = 1.00, and *β* = 1.00). However, this material is characterised by a significant degree of surface heterogeneity (*h* = 7) and a very significant development of microporous structure, as indicated by the values of the structure parameters determined by QSDFT and NLDFT methods (see Table 2).

The next materials analysed were activated carbons obtained at an activation temperature of 700 ^ο^C. The first of these, i.e. activated carbon NC-700-1, is characterised by a large volume of the first adsorbed layer (*V*_hA_ = 1.320 cm^3^/g), and only single molecules adsorb on its surface (*α* = 0.00, and *β* = 1.04). The material is also characterised by a low degree of surface heterogeneity, with a simultaneously highly developed microporous structure and a uniform adsorption energy distribution. In contrast, NC-700-2 activated carbon is characterised by geometric limitations to cluster growth, and the value of the volume of the first adsorbed layer and the structure parameters determined for this material were significantly smaller compared to the NC-700-1 sample. High clusters of adsorbate molecules form in the pores of NC-700-2 activated carbon. The analyses carried out further showed that its surface is strongly heterogeneous (*h* = 9) and the adsorption energy distribution indicates a noticeable proportion of higher adsorption energy sites on the surface of this material.

The consecutively analysed NC-700-3 activated carbon sample obtained at a mass ratio of 3 has the highest value of the volume of the first adsorbed layer (*V*_hA_ = 1.563 cm^3^/g) of all the activated carbons analysed, with single adsorbate molecules adsorbed in its pores (*α* = 0.00, and *β* = 1.00). In turn, the shape of the pore size distribution indicates a significant proportion of micropores above 1 nm in the total pore structure.

The last activated carbon sample analysed was NC-700-4, which was also characterised by a very high volume of the first adsorbed layer (*V*_hA_ = 1.542 cm^3^/g). High and unbranched clusters of nitrogen molecules were formed in the pores of this material, and its surface was characterised by the lowest degree of surface heterogeneity (*h* = 1). Structure parameters such as specific surface area and pore volume indicate the greatest development of porous structure of all the samples analyzed (*S*_QSDFT_ = 2108 m^2^/g, *V*_QSDFT_ = 1.197 cm^3^/g, *S*_NLDFT_ = 1810 m^2^/g, *V*_NLDFT_ = 1.159 cm^3^/g). The shape of the energy distribution (AED) determined for activated carbon NC-700-4 indicates a uniform distribution of adsorption energy over the surface of the material analysed, and the shapes of the pore size distributions indicate a significant proportion of micropores and small mesopores in the porous structure of this material.

The values of the micropores specific surface area *S*_NLDFT_ and the volume of micropores *V*_NLDFT_ obtained by NLDFT method, and the micropores specific surface area *S*_QSDFT_ and the volume of micropores *V*_QSDFT_ obtained via QSDFT are summarised in Table 2. Comparison of the values of the specific surface area and pore volume parameters determined using the NLDFT and QSDFT methods indicates their lower values obtained via the NLDFT method.

Based on the obtained results, it can also be concluded that the QSDFT method, which accounts for surface heterogeneity, represents a significant advancement in the characterisation of the porous structure of carbon adsorbents, including the determination of pore size distributions from adsorption isotherms, particularly in the low-pressure range when compared with the NLDFT method. This improvement is associated with the markedly increased reliability of the pore size distributions: the sharp minima appearing as artificial gaps in the NLDFT-based distributions do not occur in the pore size distributions determined using the QSDFT method.

## Conclusions

This paper presents the original results of analyses of the influence of preparation conditions on the formation of the porous structure of activated carbons dedicated to carbon dioxide adsorption obtained from coconut shells and doped with nitrogen by combining ammoxidation with KOH activation. Advanced methods of porous structure analysis were used in the analyses, i.e. the clustering based adsorption analysis process, the quenched solid density functional theory, and the non-local density functional theory methods.

Based on the results obtained, there was a significant effect of both the activation process temperature and mass ratio on the formation of the porous structure of the prepared activated carbons, and the materials with the best adsorption properties turned out to be activated carbons prepared at 700 °C with mass ratios of 2 and 3. These materials were characterised not only by the greatest development of microporous structure, but also by the lowest degree of surface heterogeneity. Significant differences were observed between the shapes of the determined pore size distributions using the QSDFT and NLDFT methods due to differences in the structure models adopted in these methods. Namely, the NLDFT method assumes that the structure is composed of smooth graphite structures, in contrast to the QSDFT method which takes into account surface roughness and heterogeneity. In conclusion, it is important to emphasise the complementarity of the analysis methods used, which together provide a wide range of information about the porous structure. As a result, the present study provided unique results, thus shedding new light on issues concerning the preparation of activated carbons and the complex characterisation of the porous structure of these materials.

## Data Availability

Data analysed during this study are included in article https://doi.org/10.1021/acs.est.5b01311, and data generated during this study are included in this published article.
